# Connecting Malfunctioning Glial Cells and Brain Degenerative Disorders

**DOI:** 10.1016/j.gpb.2016.04.003

**Published:** 2016-05-28

**Authors:** Natalie Kaminsky, Ofer Bihari, Sivan Kanner, Ari Barzilai

**Affiliations:** 1 Department of Neurobiology, George S. Wise, Faculty of Life Sciences, Tel Aviv University, Tel Aviv 6997801, Israel; 2 Sagol School of Neuroscience, Tel Aviv University, Tel Aviv 6997801, Israel

**Keywords:** DNA damage response, Genomic instability, Brain degenerative diseases, Glial cells, Astrocytes, Microglia

## Abstract

The **DNA damage response** (DDR) is a complex biological system activated by different types of DNA damage. Mutations in certain components of the DDR machinery can lead to **genomic instability** disorders that culminate in tissue degeneration, premature aging, and various types of cancers. Intriguingly, malfunctioning DDR plays a role in the etiology of late onset brain degenerative disorders such as Parkinson’s, Alzheimer’s, and Huntington’s diseases. For many years, brain degenerative disorders were thought to result from aberrant neural death. Here we discuss the evidence that supports our novel hypothesis that **brain degenerative diseases** involve dysfunction of **glial cells** (**astrocytes**, **microglia**, and oligodendrocytes). Impairment in the functionality of **glial cells** results in pathological neuro-glial interactions that, in turn, generate a “hostile” environment that impairs the functionality of neuronal cells. These events can lead to systematic neural demise on a scale that appears to be proportional to the severity of the neurological deficit.

## The DNA damage response

The most serious threat to genome stability is damage inflicted on DNA molecules [1]. Although DNA damage is usually mentioned in connection with environmental agents, in fact most of the ongoing damage to the cellular genome – estimated around tens of thousands of lesions each day – is caused by endogenous reactive oxygen species (ROS) produced during normal metabolism [1], [2]. Proper action of the defense systems that guard the genome against these ongoing threats is critically important for cellular homeostasis and development, as well as prevention of undue cell loss, premature aging, and various types of malignancies [3], [4]. These defense systems respond to DNA lesions by activating specific DNA repair mechanisms, which operate on a variety of DNA base lesions, base-pair mismatches, crosslinks, as well as single-strand breaks (SSBs) and double-strand breaks (DSBs) [5]. DNA repair is, however, just one arm of the broad DNA damage response (DDR). The DDR is an elaborate signaling network that swiftly modulates many physiological processes; it constitutes one of the most comprehensive cellular responses to physiological stimuli [6]. Recognition of the breadth and power of the DDR has come mainly from studies of the response to the DNA DSB – an extremely cytotoxic DNA lesion that vigorously activates the DDR [7].

The relationship between genome stability and human health is highlighted by the genome instability syndromes. Patients with these syndromes are typically characterized by progressive degeneration of specific tissues, chromosomal instability, cancer predisposition, and increased sensitivity to DNA damaging agents [8], [9], [10]. Predisposition to specific types of cancers can be conferred by heterozygosity for mutations that inactivate certain DDR components, such as p53, breast cancer type 1 susceptibility protein (BRCA1), breast cancer type 2 susceptibility protein (BRCA2), and mismatch repair (MMR) proteins [11], [12], [13]. These phenotypes highlight the intimate link between genome instability and the formation and progression of cancers [4], [14]. It has recently become evident that variations in DDR efficiency contribute to the development of metabolic and cardiovascular diseases [15], [16]. It is our notion that combinations of sequence alterations in DDR genes account for a continuum of variation in genome stability in the human population that affects public health on a large scale.

Mutations in key DDR molecules are implicated in human genomic instability syndromes [17]. These disorders include ataxia-telangiectasia (A-T, mutation in the *ATM* gene encoding ataxia-telangiectasia mutated) [18], ataxia-telangiectasia-like disorder (A-TLD, mutation in meiotic recombinant 11 homolog; *MRE11*) [19], Seckel syndrome (mutation in ataxia telangiectasia and Rad3 related; *ATR*) [20], and Nijmegen breakage syndrome (NBS, mutation in nibrin *NBS1*) [21]. Patients suffering from A-T, A-TLD, or NBS exhibits symptoms involving neural and lymphoid organs. A-TLD patients generally have a slower progression of disease than A-T patients do [22], [23]. NBS patients have a phenotype on the cellular level similar to A-T, except that cerebellar defects are different [21], [24], [25], [26], [27]. The phenotype of the Seckel syndrome overlaps with some features of A-T, A-TLD, and NBS (*e.g.*, microcephaly, mental retardation, genomic instability, and hematological malignancies). Patients with each of these diseases have neurological defects, suggesting that the DDR is important in neurogenesis and neurodegeneration.

## A-T – a brain degenerative disease caused by genome instability

Malfunctioning DDR is implicated in many human brain degenerative diseases (BDDs) [28] including the prototypical genome instability syndrome A-T. The hallmarks of A-T are severe neuro-motor dysfunction (emanating primarily from progressive cerebellar atrophy), telangiectasia (formation of small dilated blood vessels), immunodeficiency, sterility, a striking predisposition to lymphoid malignancies, extreme sensitivity to ionizing radiation, and, in some patients, growth retardation, premature aging, as well as insulin-resistant diabetes [29], [30], [31]. Cerebellar ataxia is one of the most devastating symptoms of A-T and progressively develops into general motor dysfunction [32]. One of the main causes of death of A-T patients is aspiration due to cerebellar-related swallowing defects [33]. Post-mortem studies reveal a significant loss of Purkinje and granule neurons in the cerebellums of young A-T patients, and therefore this disease was once considered a “Purkinje cell disease” [34], [35]. The observed damage to cerebellar neurons due to the loss of the *ATM* gene supports the “neuron doctrine” thought to underlie neurodegenerative diseases. Cellularly, A-T is characterized by cerebellar degeneration of various cell types, premature senescence of fibroblasts, chromosomal instability, and hypersensitivity to DNA-damaging agents, particularly those that induce DSBs [36]. Such increased sensitivity results from a profound defect in the cellular response to DSBs, which in normal cells chiefly mobilize ATM kinase [8].

## Malfunctioning DDR affects brain functionality

ATM deficiency is a representative of genomic instability disorders that severely affect brain functionality. Thus, we will focus on ATM deficiency and its effects on neuronal and glial cell functionality. Neurons contain significant levels of ATM in the cytoplasm [37]. The cytoplasmic ATM is found in synaptosomes, the synaptic termini of neurons, where it forms a complex with synaptobrevin (also known as vesicle-associated membrane protein 2, VAMP2) and synapsin-I. Synaptobrevin is part of a complex structure know as soluble *N*-ethylmaleimide-sensitive factor activating protein receptor (SNARE) that mediates synaptic vesicle fusion with the cell membrane during the release of neurotransmitters, while synapsin-I is an abundant neuronal phosphoprotein that is associated with synaptic vesicles [38]. Interestingly, both synaptobrevin and synapsin-I must be phosphorylated in order to bind ATM [37]. ATM deficiency leads to reduced long-term potentiation (LTP) at the Schaffer collateral-CA1 pathway, suggesting a role for cytoplasmic ATM [37].

It is highly likely that information coding in the brain is not performed at the level of single neurons but rather at the level of neural-glial networks [39]. Many BDDs are characterized by malfunctioning DDR, and it is important to understand how malfunctioning DDR influences the dynamics of neural-glial networks. Using microelectrode arrays to simultaneously record data from many neurons, Levine-Small et al. [40] analyzed how ATM deficiency affected the dynamics of neural-glial networks. Interestingly, no differences in firing activity between individual wild-type and ATM-deficient neurons were detected in response to DNA damage. In contrast, ATM deficiency led to a decrease in synchronization persistence compared to wild-type cortical networks following chemically-imposed DNA damage. These findings support the notion that neurological symptoms are not always the product of the malfunction of one cell but rather result from the failure of interacting networks. Thus, an additional implication of the study by Levine-Small et al. is that understanding the systems-level network is critically important for general comprehension of BDDs and for the development of treatment modalities for brain illness.

In a quest to understand the role of ATM in the activity of Purkinje cells, Chiesa et al. conducted morphological and electrophysiological analyses of Purkinje cells from *ATM*^−/−^ mice of different ages [41]. No histological or immunohistochemical abnormalities were found in these mice. Electrophysiological analyses revealed no abnormalities in resting membrane potential, input resistance, or anomalous rectification. However, significant reductions in the durations of calcium and sodium firing in mutant mice compared to wild-type mice were measured. The calcium deficit became significant between the ages of 6–8 and 12–20 weeks and appeared to be progressive. Voltage clamp recording showed that the firing deficits are due to a significant decrease in calcium currents, whereas the inactivating potassium currents were unaffected [41]. Taken together, these results indicate that ATM deficiency severely affects cerebellar functionality.

In addition to cerebellar defects in *ATM*-deficient mice, severe deficiencies are found in the retina of *ATM*^−/−^ mice, which affect retinal neurons, vascular system, and retinal electrical activity. Retinal neurons are terminally differentiated and have the highest metabolic activity of all neurons in the central nervous system (CNS) [42]. This makes them highly susceptible to DNA damage, which in turn severely disrupts cell metabolism. The retinal neurons are highly dependent on glial-vascular support. ATM deficiency leads to reduced integrity of the retinal vasculature, which was associated with increased levels of vascular endothelial growth factor (VEGF) and of fibrinogen [43]. In addition, ATM deficiency reduces the levels of cell adhesion molecules such as occludin and consequently leads to retinal micro–hemorrhages [43]. Morphological analyses of wild-type and ATM-deficient retinal astrocytes reveal structural alterations in *ATM*-deficient cells [43]. Amplitude aberrations were observed in 2-month-old *ATM*^−/−^ mice, which progressed to significant functional deficits in the older mice. Taken together, these findings suggest that hampered vascularization and astrocyte–endothelial cell interactions in the CNS have an important role in the etiology of A-T and that vascular abnormalities likely aggravate brain degeneration.

Brain functionality is highly dependent on astrocytes. Malfunctioning astrocytes can cause many brain diseases [44], [45] including NBS. Conditional inactivation of *NBS1* in the CNS (NBS1-Δ-CNS) leads to severe cerebellar atrophy characterized by reduced number of Purkinje cells [46]. Furthermore, there are reduced levels of cerebellar granule neurons as well as microglial cells in these mice [47]. It is of note that these mice exhibit the reduced astrocytic functionality as evidenced by reduced levels of glutamine synthetase, brain-derived neurotrophic factor (BDNF), and neurotrophic factor 3 (NT3) [47].

## Glial cells: central players in brain homeostasis and functionality

Until very recently, brain function was thought to be mainly dependent on neuronal cells. The “neuron doctrine”, which has governed modern brain research since the late 19th century [48], [49], portrayed neurons as the basic information processing unit of the nervous system, implying that neurodegenerative disorders are diseases of neurons. Because of this, most, if not all, A-T neurological research has focused mainly on Purkinje cells, granule neurons, dopaminergic neurons, but ignoring other types of CNS cells [50]. Recent studies lead to the idea that glial cells are critical to brain function. Over the course of evolution of the brain, there are many changes involving glial cells [51]. It has been estimated that protoplasmic astrocytes in the human neocortex are threefold larger in size and have tenfold more primary processes as compared to their rodent counterparts [51], [52]. Most importantly, astrocytes in higher primates display a much larger complexity as compared, for example, with those of rodents. Protoplasmic astrocytes derived from human brains manifest a threefold larger diameter and have tenfold more primary processes than those of rodents [51]. It has been estimated that every human protoplasmic astrocyte contacts and enwraps ∼2 million synapses compared to only ∼100,000 synapses covered by the processes of a mouse astrocyte [51]. Interestingly, the difference in the morpho-physiological complexity of neurons between humans and other species is relatively small. For example, the density of synaptic contacts in the brains of rodents and humans is roughly the same at around 1–1.4 × 10^9^ mm^−3^ [51]. The differences between human astrocytes and those of rodents are summarized in Table 1.

During the past 20 years, knowledge regarding the appearance, physiological properties, and functions of glial cells has grown tremendously [53]. Accumulating evidence clearly shows that glial cells (and especially astrocytes) are as diverse as neurons. The micro-architecture of the brain matter is shaped by glial cells [54]. Many of the receptors and channels expressed in glial cells are functionally similar to those of neurons. Glial cells can release gliotransmitters, enabling them to form communicating networks capable of long-range information exchange. They also respond to synaptic inputs; and, finally, they can act as pluripotent neural precursors for adult neurogenesis [55]. Astrocytes are active partners in synaptic function, integrating and processing synaptic information, as well as influencing synaptic transmission and plasticity [56]. The current understanding of gliobiology constitutes a challenge to the “neuron doctrine”, and fundamentally reshapes our perception of brain organization, which undeniably will lead to a more inclusive theory of brain function.

For many years BDDs have been thought to be primarily associated with dysfunction or death of neural cells. However, emerging evidence points to dysfunction in neuron-glia communications as playing a large role [57]. Indeed, it appears that glia cells (astrocytes in particular) are much more than just the “glue” that holds together the neurons of the CNS. Rather, glia are very active participants in the neuronal network. Glia can release “gliotransmitters” and have many of the same receptors as neurons, indicating that glia and neurons can speak and comprehend the same chemical language [54]. Accordingly, it no longer makes sense to consider networks of neurons in isolation, since neurons and glia act in concert to form the circuits and networks of the CNS. Given the importance of neuron-glia communications, and the insights stemming from a large body of previous studies, it is reasonable to assume that dysfunctional neuron-glia communications play a major role in BDDs that result from a malfunctioning DDR.

**Figure 1:**
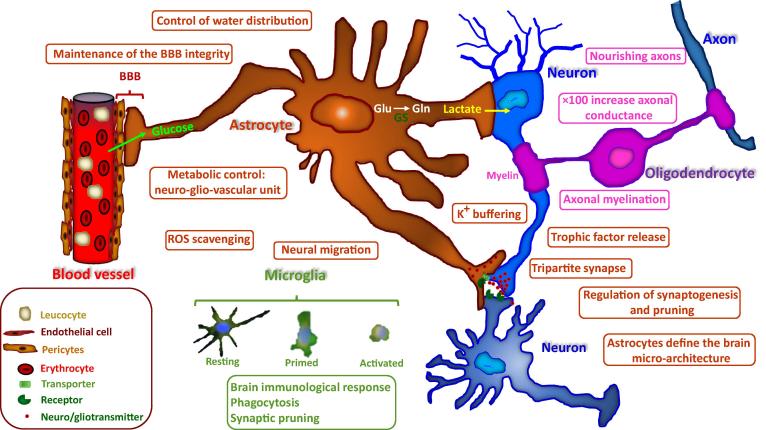
**The physiological roles of glia cells** Brain homeostasis is critically dependent on the functionality of the glial cells. As depicted in the figure and as described in the text, astrocytes are involved in many brain functional processes such as the regulation of the vascular system and the blood–brain barrier, metabolic control, maintenance of homeostatic redox state and buffering of electrolytes, as well as control of water distribution. In addition, astrocytes control synaptogenesis and synaptic pruning. Microglial cells control brain immunological responses, phagocytose dying cells as well as various debris, and are responsible for synaptic pruning through the complement system. Oligodendrocytes regulate axonal myelination. BBB, brain blood barrier; GS, glutamine synthetase; ROS, reactive oxygen species.

Astroglial cells function under both normal and pathological conditions. Under normal physiological conditions, astroglial cells control water movement and distribution, as well as buffer the concentrations of extracellular potassium ions. They are capable of regulating the release of neurotrophic factors as well as extracellular concentrations of neurotransmitters released from neighboring synapses. One of the most important roles of glial cells is the neutralization of ROS generated in the vicinity of various types of cells including neurons. Astrocytes are important for metabolic support of adjacent neurons. They do so by absorbing glucose from blood vessels that they contact and converting it to lactate, which is then provided to neurons (Figure 1). The functionality of astrocytes is impaired under pathological conditions. Stressed astrocytes can no longer control water movement and as a consequence can lead to brain edema. Malfunctioning astrocytes can also generate toxic levels of ROS such as hydroxyl radicals, nitric oxide, and peroxynitrite [57], [58], [59].

Astrocytes are also involved in various aspects of synaptogenesis, synaptic maturation, neurotransmitter homeostasis, brain microcirculation, brain metabolism, as well as control over formation and maintenance of the blood–brain barrier [60], [61], [62], [63], [64], [65], [66], [67], [68], [69], [70], [71], [72], [73]. In addition, ‘sick or stressed’ astrocytes can reverse the direction of certain neurotransmitter transporters, thereby releasing substances such as glutamate, concomitant with secretion of cytotoxic levels of Ca^2+^. Increased intracellular Ca^2+^ levels can activate calcium-dependent cysteine proteases (calpains), thereby causing neural demise [74]. Failure to buffer extracellular K^+^ concentrations promotes further over-excitation of neuronal cells [58]. Stressed astrocytes can affect the aforementioned processes. Like neurons, astrocytes can communicate with surrounding cells through plasma membrane channels, transporters, receptors, and certain molecules and by undergoing exocytosis or endocytosis [75], [76], [77], [78], [79], [80], [81].

## The effects of DNA damage on astroglial cell functionality

Whereas neuronal cells are highly differentiated and post-mitotic cells, mature astrocytes in the brain are capable of cell proliferation [82], [83]. As mitotic cells, one would assume that DNA damage in astrocytes could be repaired through either homologous recombination (HR) or non-homologous end joining (NHEJ), depending on the mitotic phase of the cell. Adams et al. measured the rate of DNA DSB repair in human embryonic stem cells (hESCs), neural progenitors, and astrocytes. They showed that generation of γH2AX in hESCs was slower than in neural progenitors and astrocytes, suggesting that DSB repair in hESCs is more complex compared to that in neural progenitors and astrocytes [84]. It seems that the activity of HR repair is highest in hESCs and declines as a function of differentiation with little activity detected in neural progenitors and no trace of activity detected in astrocytes. Interestingly, ATM plays a major role in NHEJ and a minor role in HR repair in stem cells. These results may provide an explanation regarding the progressive nature of A-T and why the absence of ATM does not lead to embryonic lethality. ATM seems to be very important for DSB repair in fully-differentiated astroglial cells [85]. ATM deficiency in the brain impairs the functionality of the neuro-glio-vascular unit.

Unlike mature and fully-differentiated astrocytes, glioblastoma cells retain their ability to activate HR repair. There is a dose-dependent increase of Rad51 focus formation in response to the induction of DSBs in glioblastoma cells. Down-regulation of *Rad51* expression using RNAi is associated with increased cell death following ionizing radiation (IR) treatment [86]. Schneider et al. [85] compared DDR activation and radio-resistance between somatic stem cells and terminally-differentiated descendant astrocytes. They showed that somatic neural stem cells display robust activation of the canonical DDR following exposure to IR as measured by the formation of phosphorylated ATM nuclear foci and the phosphorylation of ATM substrates p53 and γH2AX. *In vitro* and *in vivo* observations revealed that compared to somatic stem cells, ATM activation and its downstream DDR factors such p53, checkpoint kinase 2 (CHK2), and ATR are strongly inhibited in astrocytes compared to somatic stem cells. The inhibition of the DDR machinery involves a stable transcriptional repression of key DDR genes as mentioned above. The astroglial cells examined in this investigation are non-proliferative mature terminally-differentiated cells, mimicking the state of most of adult astroglial cells. Interestingly, astrocytes retain the expression of NHEJ genes and are proficient in DNA repair. It seems that upon DNA damage induced by IR, DNA-dependent protein kinase DNA-PK, a PI3K-like kinase, is responsible for γH2AX signal in astrocytes [85]. ATR plays similar roles as DNA-PK in neuronal stem cells [84]. Together, these results suggest that at different developmental stages, ATM, ATR and DNA-PK are all responsible for H2AX phosphorylation in response to IR.

FOXO3a, the main FOXO transcription factor isoform expressed in astrocytes, is responsible for the cellular response to oxidative stress. This is achieved by regulating the expression of specific genes, including those genes encoding factors involved in DNA repair and glutamine metabolism such as glutamine synthetase. Immunohistochemical analyses of human samples from the population-based neuropathology cohort of the Medical Research Council’s Cognitive Function and Ageing Study have shown that the FOXO3a nuclear retention significantly correlates with DDR and with glutamine synthetase expression by astrocytes [87]. Moreover, a correlation between the expression of glutamine synthetase and Alzheimer’s disease-type pathology in this aging cohort was observed. Taken together, these findings suggest that in astrocytes glutamine synthetase serves a neuroprotective mechanism and is affected by nuclear retention of FOXO3a in response to oxidative stress [87].

Using a variety of cellular, molecular, and genomic methodologies, Yong et al. [88] showed that IR-induced DNA damage repair pattern at the gene locus-specific level and genome-wide level changes along with the alteration of transcriptional state of human astrocytes. Interestingly, following IR, the activity of DNA repair in actively-transcribed chromatin is increased in reactive astrocytes, compared to non-reactive astroglial cells. The authors mapped the repair sites and identified misrepaired events and repaired hotspots that were unique to each transcriptional state. In addition, Swain and Rao evaluated the DNA damage at the single-cell level in isolated neurons and astrocytes derived from rats of different ages [89]. They found the increased DNA damage in both neurons and astrocytes with advancement of age, in view of SSBs, DSBs, 8-oxoguanine (8-oxoG), as well as sites sensitive to 8-oxoG glycosylase (OGG1) and uracil DNA glycosylase. Furthermore, the authors provided evidence for age-dependent decreases in the activities of DNA repair enzymes of the base excision repair (BER) pathway. Collectively, these results show that aging affects both neurons and astrocytes through the decline of many cellular repair mechanisms.

Phosphatase and tensin homolog (PTEN) acts as a tumor suppressor [89]. Unlike most of the protein tyrosine phosphatases, PTEN preferentially dephosphorylates phosphoinositide substrates such as phosphatidylinositol (3,4,5)-triphosphate (PIP_3_) resulting in PIP_2_. PTEN is involved in the regulation of the cell cycle, preventing cells from growing and dividing too rapidly [89]. In addition, PTEN has a role in DSB repair. Exposure of *PTEN*-null astrocytes to *N*-methyl-*N*′-nitro-*N*-nitrosoguanidine, a functional analog of temozolomide, results in the accumulation of DSBs due to inefficient DNA repair and apoptosis [90]. *PTEN* deletion also compromises HR repair. It is possible that PTEN inactivation increases astrocyte proliferation and that the HR repair pathway is activated in proliferating astrocytes, although it is inactivated in non-dividing astrocytes. The compromised HR repair in *PTEN*-deficient astrocytes can be explained by reduced expression Rad51 paralogs.

## Glial cell alterations in primary cultures derived from *ATM*^−/−^ cerebella

Glial fibrillary acidic protein (GFAP) is an astrocyte-specific marker. Compared to wild type, there is a less complex cell arborization in *ATM***^−/−^** cerebellar neural-glial networks as revealed by GFAP staining; the number of branches originating from the cell bodies is also significantly lower in astrocytes of *ATM***^−/−^** mice [44]. Additionally, there is a marked reduction in GFAP expression in cerebellar sections from ATM-deficient mice, suggesting that ATM deficiency leads to reduced levels of valete astrocytes *in vivo*. Similarly, morphological alterations in astrocytes are found in ATM-deficient retinas and optic nerves. Reduced arborization of the retina and optic nerve is associated with reduced retinal functionality as shown by electroretinographic examination [43]. These findings suggest that impaired astrocytic functionality in the CNS plays an important role in the etiology of A-T.

## The functional role of microglia in health and disease

In the year 1919, Rio Hortega described a new type of glial cell, the microglia [91]. These cells descend from the hematopoietic system, invade the brain early during development, and become the resident immune system of the CNS. Nonetheless, not much was known about their functionality in health and disease for decades. In the past 10 years, our knowledge about microglia functionality has been tremendously increased due to thorough studies of various brain disorders as well as injuries [92]. Our current view of microglial cells is that these cells are major players in the brain defense system against various types of biological threats as well as injuries [93]. Morphologically, microglia behave like chameleons and change their forms from a resting state with many processes to an activated macrophage-like state (ameboid) in response to a variety of stimuli [94]. In a state of injury or disease, and following the secretion of various chemokines by astrocytes, microglial are swiftly recruited to the damage sites where they phagocytose or engulf various types of cellular debris and remove dying or unnecessary cells. In addition to their immune response to trauma or infection, microglial cells are capable of secreting neurotoxic proteins and cytokines that contribute to pathological inflammation [95], [96]. In 2010, Ginhoux et al. showed that microglial cells develop from primitive myeloid progenitors that arise before embryonic day 8 to invade the brain [97]. These findings challenge the current dogma that microglia develop from peripheral macrophages. The data reported by Ginhoux et al. suggest that the microglia are ontogenically a population of mononuclear phagocytic cells [97]. New evidence implicates microglia in building and wiring of the developing CNS, through functions that range from neurogenesis to synaptic pruning [98], [99].

## DDR in microglia

Alkylating agents, such as methylnitrosourea (MNU), severely affect mitochondrial DNA (mtDNA). In response to MNU, there are no cell-specific differences in initial mtDNA damage repair in different glial cells including microglia, astrocytes, and oligodendrocytes. However, compared to astrocytes, mtDNA repair in oligodendrocyte progenitors, oligodendrocytes, and microglia was significantly reduced. A variety of xenobiotics can lead to mitochondrial perturbations, which would culminate in apoptosis. Whereas MNU-treated oligodendrocytes display DNA laddering and other characteristics of apoptosis, no signs of apoptosis are detected in astrocytes exposed to MNU [100].

Carcinogenesis and neurodegeneration can be caused by ROS lesions that lead to the formation of 8-oxoG. Severe striatal neurodegeneration is detected in mice lacking genes encoding 7,8-dihydro-8-oxoG triphosphatase (MTH1) that prevents the incorporation of 8-oxo-2′-deoxyguanosine triphosphate to the DNA and/or OGG1 that excises 8-oxoG. In contrast, mice deficient in mutY homolog (MUTYH) and/or OGG1 are resistant to neurodegeneration induced by oxidative stress. Together, these findings indicate that MUTYH, a glycosylase involved in oxidative DDR, promotes neurodegeneration, whereas OGG1 and MTH1 are protective. Accumulation of 8-oxoG in neuronal mtDNA leads to calpain-dependent neuronal loss. This process is associated with delayed 8-oxoG nuclear accumulation in microglial cells leading to PARP-dependent activation of apoptosis-inducing factor and exacerbated microgliosis. Taken together, these findings show accumulation of 8-oxoG in the genomes of neurons and microglia results in neurodegeneration [101].

Alterations in microglia have been suggested to contribute to age-related CNS deterioration. Enhanced sensitivity to various inflammatory stimuli is among the most prominent age-related changes in microglial cells, which is referred to as priming. During microglial priming, microglia multiply and adopt an activated state in response to neurodegeneration and abnormally folded proteins [102]. Whether microglial cell priming is an intrinsic microglial aging process or induced by the aging neural environment is not clear. DNA excision repair protein 1 (*Ercc1*) mutant mice, which display DNA repair deficiencies, serve as a good model for accelerated aging. These mutant mice show the accelerated aging in various tissues including the brain. Hallmarks of microglial priming such as cytokine expression and phagocytosis can be detected in the *Ercc1* mutant mice in response to peripheral lipopolysaccharide. Progressive priming response of microglial cells is also observed in mice with *Ercc1* deleted in forebrain neurons, in association with phenotypic alterations [103].

In human Alzheimer’s disease patients, elevated S-adenosylhomocysteine (SAH) is found in plasma and cerebral tissue, which is inversely correlated with the cognitive ability of the patients [104]. Moreover, treatment of BV2 cells (mouse immortalized microglia) with SAH causes DNA hypomethylation, irreversible DNA damage, and cytotoxicity [105]. This appears to be attributed to the inhibition of DNA methyltransferase 1 (DNMT1) by SAH. Interestingly, exposure of the BV2 cells to SAH also leads to increased level of amyloid beta, which then lead to the generation of ROS, increased levels of 8-oxoG, and reduced expression of OGG1 [106].

(+)-Catechin, a flavanol antioxidant extracted from Acacia catechu [107], efficiently inhibits tert-butylhydroperoxide-induced DNA damage and cell death. Huang et al. have shown that down regulation of p53 phosphorylation following (+)-catechin treatment decreases the levels of hydroxyl radicals and cell cycle arrest. The reduced p53 activity follows the impairment of NF-κB translocation to the nuclear region [108].

## NBS1 inactivation compromises the functionality of astrocytes

Hypomorphic mutations in the *NBS1* locus lead to the mental retardation, microcephaly, radiation sensitivity, chromosomal instability, immunodeficiency, and cancer predisposition experience by patients with NBS [21], [27]. The MRN complex is formed by the association of Mre11, Rad50, and NBS1. The complex is involved in sensing cell cycle checkpoints and DSBs [109]. Early embryonic lethality is observed *NBS1* – null mice, making it difficult to study the physiological functions of NBS1 during development [110], [111].

We hypothesized that *NBS1* deletion specifically in astrocytes would affect the DDR and thereby cause cerebellar pathology and behavior. We generated the GFAP-Cre-lox mouse strain with down-regulated expression of the NBS1 specifically in astrocytes (Binyamini H et al., in preparation). These NBS1-GFAP-Δ astrocytes displayed reduced functionality as well as malfunctioning DDR in response to X-ray irradiation or the radiomimetic agent neocarzinostatin. Compared to wild-type cells, expression of BDNF and glutamine synthetase (GS) is reduced in the *NBS1*-deficient astrocytes. Interestingly, there is no evidence of altered cerebellar morphology in 10 month-old NBS1-GFAP-Δ mice, which seem to have normal behavior. Therefore, the observed malfunction DDR may not be sufficient to cause pathological effects. It is also possible that *NBS1* deletion in one cell population is not sufficient to cause cerebellar pathology (Binyamini H, personal communication).

## Reduced microglia recruitment results from conditional deletion of NBS1 in the CNS

*CD11b* encodes an integrin expressed specifically in microglia [47]. *CD11b* expression is significantly reduced in NBS1-CNS-Δ cerebella, together with reduced levels of cysteine–cysteine chemokine ligand 2 (CCL2), the chemokine primarily responsible for attracting CD11b-positive cells and mediating the innate immune response in the CNS [112]. Other than that, expression of the gene encoding cysteine–cysteine chemokine receptor 2 (CCR2) is significantly downregulated in NBS1-CNS-Δ cerebella in comparison with wild-type mice [47]. It is of note that a *CCR2-*deficiency accelerates the severity of BDDs and markedly impairs microglial cell accumulation in the mouse brain [113].

## Malfunctioning DDR affects brain functionality via glial cell impairment

How does malfunctioning DDR affect glial cell functionality and thereby network dynamics? Glial cells are key players in neuronal homeostasis through the following processes, including metabolic support [114], [115], [116], regulation of neuro- and gliotransmitters uptake and release, secretion of neurotrophic factors, synaptogenesis, synaptic maintenance, and synaptic pruning [71], [117], [118], [119] (Figure 1).

Network dynamics is heavily dependent on network topology, the connections among neural cells, the interactions between neurons and glial cells, and on the mode of synaptic transmission. Malfunctioning DDR severely affects the glial cell functionality, and malfunctioning astrocytes can alter synaptic activity, thereby affecting the dynamics of neural-glial networks. Alterations in K^+^ ion buffering and water distribution can also affect neural activity, influencing network dynamics. The excitatory neurotransmitter glutamate and the supply of glutamine to surrounding neural cells are also important for network functionality. Our results clearly demonstrate that *NBS1* deletion reduces the secretion of neurotrophic factors and reduces expression of glutamine synthetase, the enzyme responsible for conversion of glutamate to glutamine [47]. In addition to astrocytes, microglial cells are also vital for brain functionality. Malfunctioning DDR reduces the ability of astrocytes to recruit microglial cells, thereby stripping the brain of an important protective layer. We have shown that glial cells are capable of controlling a variety of processes that can affect network dynamics. Based on the available data, we still do not know, however, how glial-controlled processes affect network dynamics.

## Closing remarks

**Figure 2:**
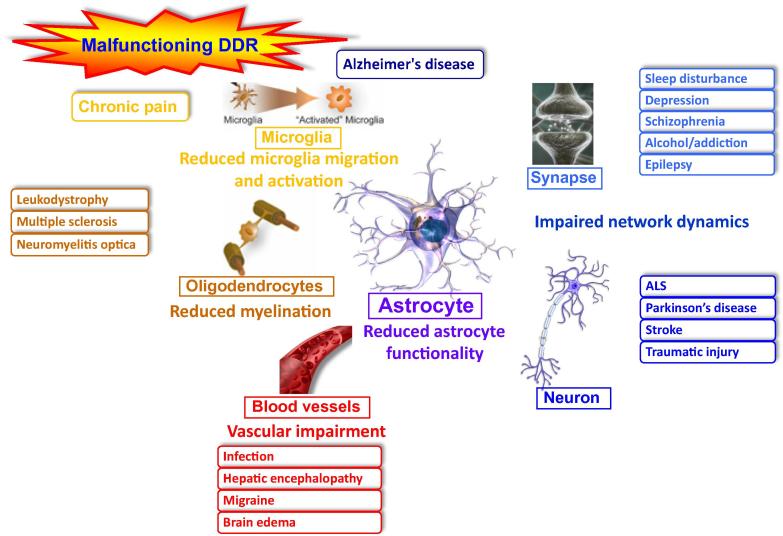
**The role of glial cells in brain degenerative diseases** Malfunctioning glial cells are involved in a variety of brain diseases as depicted in the figure and the text. DDR, DNA damage response; ALS, amyotrophic lateral sclerosis. The figure was drawn based on the data presented previously [57].

The brain is the most complicated tissue in higher organisms, responsible for control of vital processes and also at the center of motor functions, cognition, and personality. Loss of brain functions through trauma or aging exacts an enormous human toll on patients and families. Cases of age-related dementia have increased as average life spans have reached record highs. Moreover, advances in modern medicine have enabled us to replace or repair almost all body organs except the brain. To ensure that brain functionality can be maintained in old age, it is critically important to gain a better understanding of the cellular and molecular processes involved in the brain dysfunction that can result from BDDs (Figure 2).

For many years the neuronal doctrine has dominated the field of brain research, but it is now clear that various types of glial cells are critical to brain function. Like neurons, the glial cell population contains a vast variety of cells, each with a different role. Glial cells are important in the most rudimentary synaptic processes and in the regulation of complex brain networks and circuitries. Some glial cells provide the structure necessary for CNS development, others provide nutrients to support neurons, and others appear essential to activities such as the formation of synaptic connections and pruning. Some glial cells undertake immune system functions, such as the destruction of invading pathogens. Such an important role is performed by the microglial cells that invade the brain during embryonic development.

As mentioned in this review, much of the gliobiology research has focused upon astrocytes and microglia, which have very broad portfolios of responsibilities and contributions. These two populations of glial cells form highly-complex connections, which are disrupted during brain pathologies. Structurally, astrocytes are very complex and their activation has been reported in a variety of brain diseases and injuries. The specific functions of astrocytes under physiological as well as in pathological conditions are still not completely understood. This is due in part to the enormous heterogeneity of glial cells starting from stem cells and ending in mature highly polarized and differentiated cells. Naturally, this makes the comparison of individual studies rather difficult [120].

A common denominator in various BDDs is malfunctioning of DDR pathways. Here we present evidence that impaired DDR severely affects the functionality of glial cells and underlies the symptoms of certain degenerative diseases of the brain. Based on a wealth of data, we suggest that glial cells play a major role in brain aging as well as in the etiology of early onset genomic instability disorders and late onset BDDs.

## Competing interests

The authors declare that they have no competing interests.

## Figures and Tables

**Table 1:** Evolutionary alterations in human astrocytes compared to rodents

**Glial parameter**	**Rodent**	**Human**
Glia to neuron ratio	1	∼4.1-fold greater
Number of processes	1	∼10-fold greater
Process length (protoplasmic)	1	∼2.6-fold greater
Process diameter	1	∼2.9-fold greater
Cell diameter (protoplasmic)	1	∼2.6-fold greater
Cell diameter (fibrous)	1	∼2.14-fold greater; similar in shape
Cell volume (protoplasmic)	1	∼27-fold greater; much more complex
Number of supported synapses	1	∼20-fold greater
Propagation of calcium waves	1	∼4-fold faster
Average area of overlap domains	1	∼2.8-fold greater
Summated area of overlapping domains	1	∼3.8-fold greater

*Note:* The table was generated based on the data presented previously [51], [52].
